# Intestinal epithelium penetration of liraglutide via cholic acid pre-complexation and zein/rhamnolipids nanocomposite delivery

**DOI:** 10.1186/s12951-022-01743-9

**Published:** 2023-01-16

**Authors:** Xiaoyan Bao, Kang Qian, Mengjiao Xu, Yi Chen, Hao Wang, Ting Pan, Zhengyi Wang, Ping Yao, Li Lin

**Affiliations:** 1 School of Pharmaceutical Sciences, Wenzhou Medical University, Gaojiao Zone, Wenzhou, 325035 China; 2 Key Laboratory of Smart Drug Delivery, Ministry of Education, School of Pharmacy, Fudan University, 826 Zhangheng Road, Shanghai, 201203 China; 3 State Key Laboratory of Functions and Applications of Medicinal Plants, Guizhou Medical University, Guiyang, 550014 China; 4 State Key Laboratory of Molecular Engineering of Polymers, Collaborative Innovation Center of Polymers and Polymer Composite Materials, Department of Macromolecular Science, Fudan University, 2005 Songhu Road, Shanghai, 200438 China

**Keywords:** Oral, Liraglutide, Cholic acid, Rhamnolipids, Type 2 diabetes

## Abstract

**Background:**

Oral administration offered a painless way and improved compliance for diabetics. However, the emerging GLP-1 analog peptide drugs for diabetes primarily rely on the injection route, and the development of oral dosage forms was hampered by the low oral bioavailability due to the structural vulnerability to digestive enzymes and molecule impermeability in the gastrointestinal tract.

**Results:**

In this study, the non-covalent interaction between cholic acid (CA) and liraglutide (LIRA) was found and theoretically explained by molecular docking simulation. Formation of this physical complex of liraglutide and cholic acid (LIRA/CA Complex) reduced the self-aggregation of LIRA and accelerated intestinal epithelium penetration. By the anti-solvent method, LIRA/CA Complex was loaded into zein/rhamnolipids nanoparticles (LIRA/CA@Zein/RLs) with a loading efficiency of 76.8%. LIRA was protected from fast enzymatic degradation by the hydrophobic zein component. Meanwhile, Rhamnolipids, a glycolipid with surface activity, promoted endocytosis while also stabilizing the nanoparticles. The two components worked synergistically to ensure the delivery of LIRA/CA Complex to intestinal villi and improved oral absorption without disrupting tight junctions. LIRA/CA@Zein/RLs demonstrated a considerable intestinal epithelium absorption in mouse gastrointestinal section and a retention *in vivo* over 24 h, resulting in a significant and long-lasting hypoglycemic effect in Type 2 diabetes mice.

**Conclusion:**

This study provided a promising oral delivery approach for LIRA and exhibited the potential for further translation into clinical application.

**Supplementary Information:**

The online version contains supplementary material available at 10.1186/s12951-022-01743-9.

## Background

Diabetes mellitus is one of the most widespread chronic diseases in the world, and the majority is Type 2 diabetes (T2DM) caused by insulin resistance [1, 2]. There have been plenty of anti-diabetic drugs in clinic for glycemic control, but it is still hard to simultaneously meet all the requirements, including satisfactory blood glucose level management, low side effects, and high patient compliance [3]. The discovery of glucagon-like peptide-1 receptor agonists (GLP-1 RAs) brings some changes in guidelines on T2DM management. These agents stimulate insulin release in a glucose-dependent manner and suppress glucagon activity during hyperglycemia [4]. The lower risk of hypoglycemia and weight loss effects make GLP-1 RAs competent choices in T2DM treatment [5].

Considering the lifelong treatment, oral administration is one of the most attractive routes which leads to earlier initiation of treatment and improved adherence. Oral absorption of GLP-1 RAs mimics the physiological route of endogenous GLP-1 [6], which is secreted by L-cells in the distal ileum and then enters the blood circulation [7]. However, owing to the hostile environment of the gastrointestinal tract (GIT) and the challenges from molecules’ size and polarity, the oral delivery of proteins and peptides is hampered by enzyme degradation and low permeability [8].

In the attempt to improve oral absorption of macromolecular drugs, absorption enhancers are commonly used because of their availability and practicability. However, the overall absorption promotion of macromolecules by absorption enhancers was still limited [9], which could be ascribed to absorption enhancers’ inability to offer protection against enzymes and potential toxicity in long-term administration. [10, 11] In September 2019, the oral semaglutide tablet entered the market, in which, semaglutide forms a physical complex with the absorption enhancer sodium N-[8-(2-hydroxybenzoyl) amino] caprylate (SNAC) [12]. Interestingly, besides the traditional effects of changing tight junctions (TJs) or membrane fluidization, the complex of semaglutide and SNAC protects semaglutide against pepsin and augments a concentration-dependent transcellular flux of semaglutide *via* increasing its solubility through molecular interaction with SNAC. Thus, the absolute oral bioavailability of semaglutide tablets was improved to about 0.4–1% [13]. Liraglutide (LIRA), also as a fatty acid-acylated GLP-1 analog, not only improves glycemic control but also leads to weight loss and cardiovascular benefits [14, 15]. The once-daily subcutaneous LIRA is effective for blood glucose control but not favorable for the earlier initiation of treatment, thus the development of oral LIRA dosage forms is appealing. However, the SNAC complexation strategy mentioned above is highly specific and limited to semaglutide [12]. As reasonable speculation, an appropriate absorption enhancer forming LIRA complex that is analogous with semaglutide/SNAC complex could translate into better oral LIRA absorption.

As absorption enhancers for poorly permeable molecules, cholic acid (CA) and its derivatives as endogenous molecules have been widely tested [16]. They commonly enhance the absorption by three approaches: formation of a micelle, incorporation into the liposomes, and conjugation with the drug molecules or groups on the particle surface [17, 18]. These CA or CA derivative-containing nanoparticles (NPs) could achieve transcytosis by bile acid transporter in the apical membrane of the intestinal milieu at high efficiency. [18] Besides, the glycoprotein depletion ability of CA could reduce the trap of NPs in adhesive mucus [19]. However, the direct forming of a physical complex between CA and peptides to improve oral permeability has not been reported.

Herein, LIRA/CA Complex-loaded Zein/RLs NPs were designed to orally deliver LIRA for the treatment of T2DM. The formation of LIRA/CA Complex by non-covalent interactions between LIRA and CA was expected to improve the oral absorption of LIRA. Given the proteases in GIT, hydrophobic protein zein was applied to protect LIRA/CA Complex. In our previous research, zein displayed excellent biocompatibility even at high doses and successfully acted as cement to embed peptides and slowed down the pepsin and pancreatin degradation [20]. However, zein NPs were easy to aggregate during preparation and storage owing to the high hydrophobicity [21]. Coating with amphiphilic or hydrophilic materials was a helpful tactic to prevent hydrophobic aggregation, [22, 23] and our earlier study suggested that selecting the proper stabilizer was essential since the highly hydrophilic chain may have delayed the endocytosis of zein NPs [24]. Rhamnolipids (RLs) as surface-active glycolipid can reduce the hydrophobicity of zein NPs, [25] thereby improving the aggregation stability [26]. Besides, amphiphilic RLs were reported to increase the epithelial permeability of macromolecules by enhancing both paracellular and transcellular transport, but the mechanism behind this still requires investigation. [27].

**Scheme 1:**
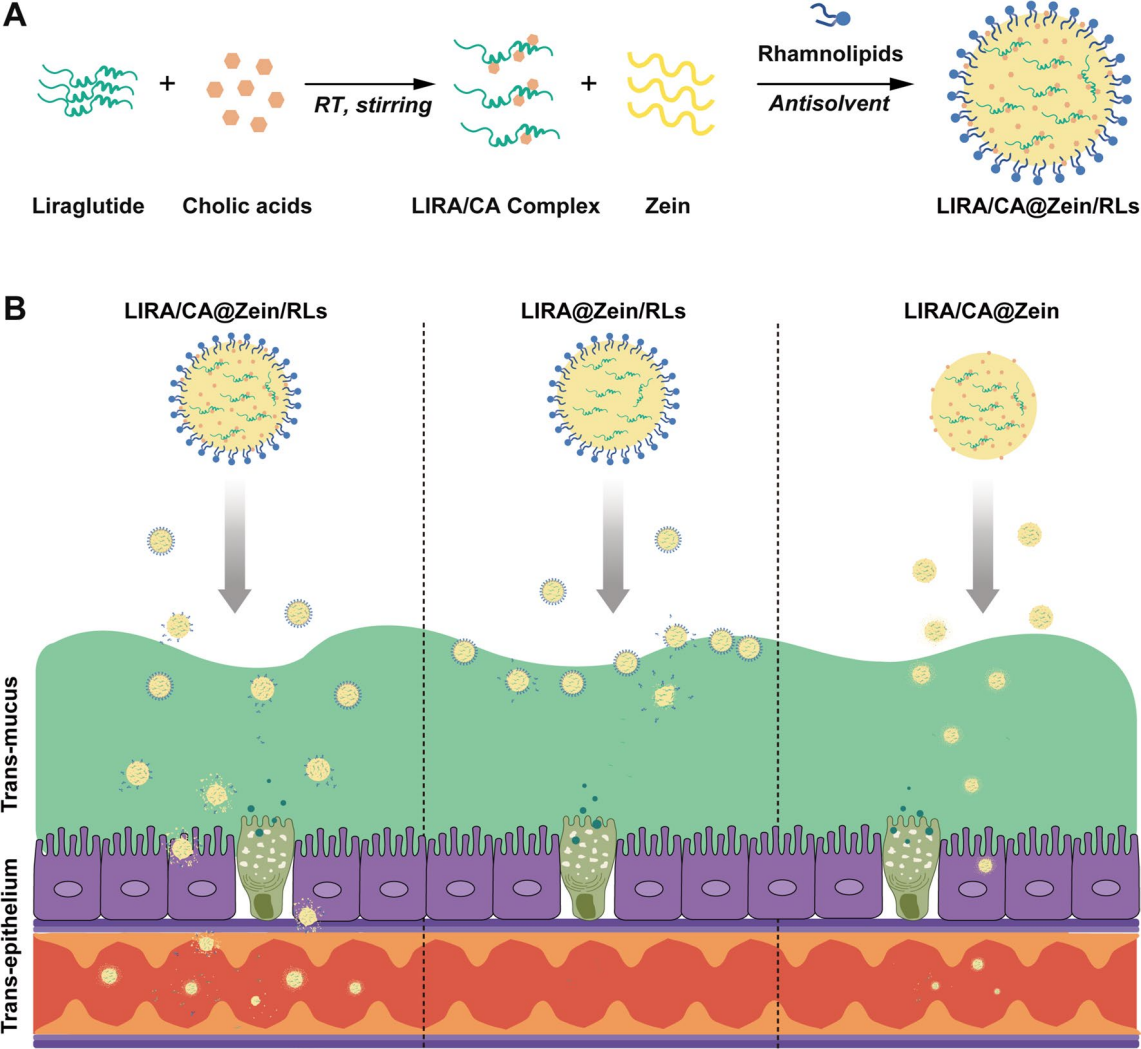
Schematic illustration of the nanocomposites (LIRA/CA@Zein/RLs) accelerating LIRA oral absorption by facilitating trans-mucus and trans-epithelial permeability. **A** The preparation of LIRA/CA@Zein/RLs by self-assembly of zein using anti-solvent precipitation method. **B** In the gastrointestinal tract, zein, CA, and RLs worked cooperatively: zein slowed down the pepsin and pancreatin degradation; CA could enhance trans-mucus and trans-epithelium efficiency of LIRA when RLs mainly stabilized the NPs and improved transcytosis

In this study, LIRA/CA@Zein/RLs was prepared by anti-solvent precipitation method, which exhibited high encapsulation efficiency and ease of scale-up. As shown in Scheme 1, the absorption enhancer CA, stabilizer RLs and carrier zein were expected to transport LIRA cooperatively across the intestinal epithelium. LIRA/CA Complex was characterized by FTIR and the molecular interaction was simulated by molecular docking. We also investigated the transcellular permeability of LIRA/CA@Zein/RLs in vitro and in vivo. The systemic absorption in healthy mice and the hypoglycemic effect in T2DM mice were evaluated in vivo.

## Results and discussion

### Interactions between liraglutide (LIRA) and cholic acid (CA)

**Fig. 1:**
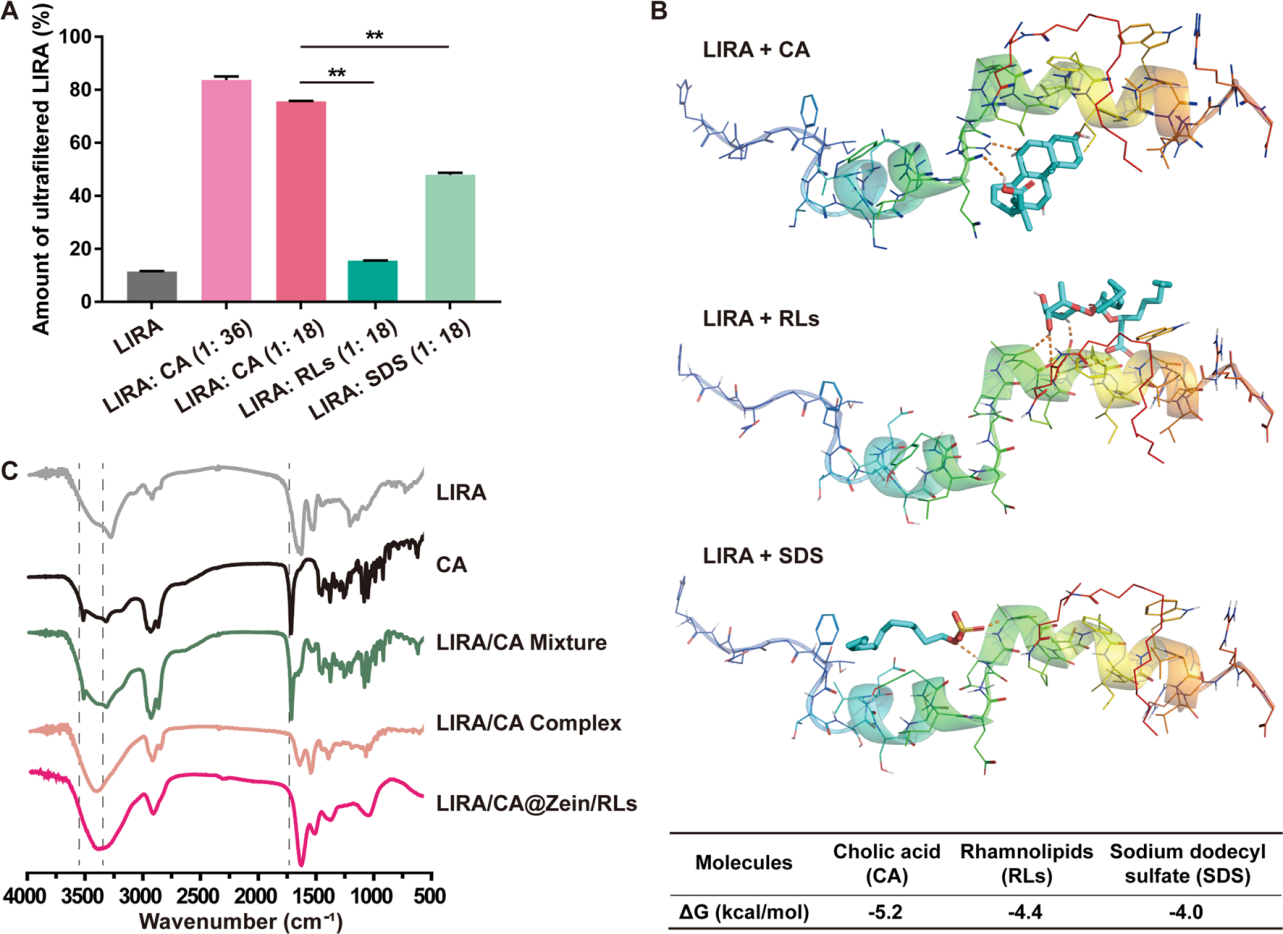
Characterization of LIRA/CA Complex. **A** The penetration rate of free LIRA and LIRA complexed with CA, RLs, or SDS after ultrafiltration by the membrane with a cutoff molecular weight of 50 kDa. **P < 0.01, compared with the LIRA: CA (1: 18) group (n = 3). **B** The images and computational binding free energies of the best docking conformations between LIRA and ligands (CA, RLs, and SDS) with the highest binding affinity obtained by Autodock Vina. Hydrogen bonds were marked by red dotted lines. **C** FTIR spectra of LIRA, CA, physical mixture of LIRA and CA, LIRA/CA Complex, and LIRA/CA@Zein/RLs

Due to the palmitoylation, LIRA could self-assemble into oligomers [28]. And such a high-molecular oligomeric form of LIRA could not freely pass through the pores in the membrane of the ultrafiltration cell, which correspondingly resulted in the low ultrafiltration rate of pure LIRA solution (Fig. 1 A). Interestingly, the addition of CA significantly increased the ultrafiltration rate of LIRA from 11.5 to 75.6% (LIRA: CA = 1: 18), suggesting the dissociation of the LIRA oligomers induced by the interaction between LIRA and CA. By comparison, the impacts of sodium dodecyl sulfonate (SDS) and RLs on LIRA oligomers were also investigated, and with the same molar ratio, the ultrafiltration rate of LIRA in the CA group was 1.6- and 4- fold of that in SDS and RLs groups.

To further clarify the interaction between LIRA and CA. Molecular docking *via* Autodock Vina was explored to obtain their binding pose and affinity. As shown in Fig. 1B and Additional file 1: Fig. S1, CA has the lowest binding free energy, in other words, possessing higher affinity with LIRA than SDS and RLs. The binding position of CA in the best docking conformation was at the linker position between the two α-helix domains, which was different from the binding positions of RLs and SDS. The results of molecular docking could also point out the location of hydrogen bonds between LIRA and absorption enhancers. In the best docking conformation of CA and LIRA, the hydrogen bonds were formed by the carboxyl and hydroxyl groups of CA with the carboxyl and amino groups of amino acid residues at position 21 (glutamic acid) and 23 (glutamine) of LIRA from the N-terminal, respectively. To better understand the non-covalent interaction between LIRA and CA, Fourier transform infrared spectroscopy (FTIR) spectra were acquired and shown in Fig. 1 C The spectra of individual CA and LIRA were consistent with references [29, 30]. Compared with the FTIR spectrum of the physical mixture of LIRA and CA, an interaction between LIRA and CA in LIRA/CA complex was indicated by the peak shifts and intensity changes of the C = O of COO^−^ stretching band at 1715 cm^− 1^ and the O-H stretching vibrations at 3853 and 3321 cm^− 1^, which was in good agreement with the molecular docking result.

### Preparation and characterization of LIRA-loaded nanoparticles

**Fig. 2:**
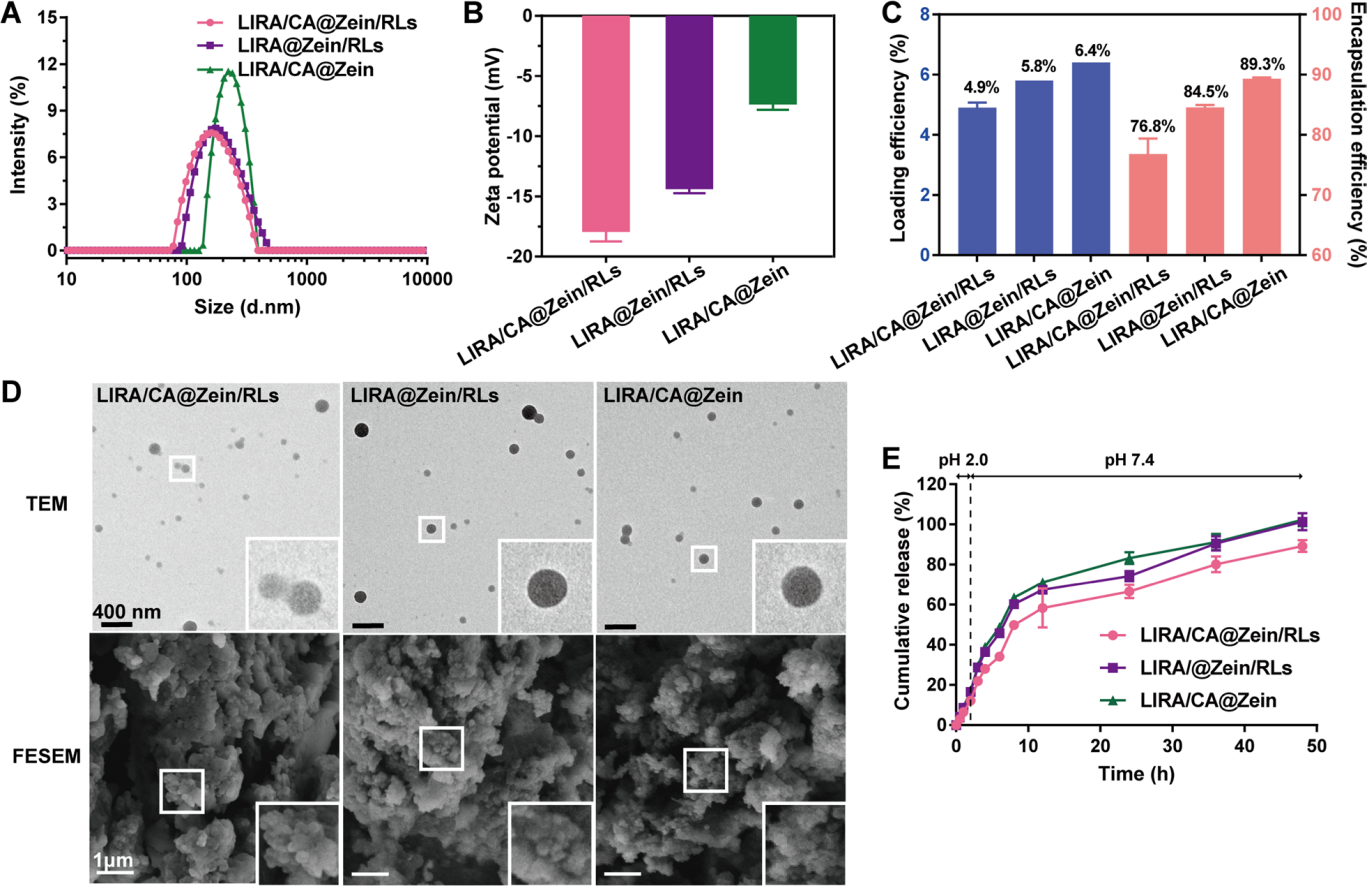
Characterization of LIRA-loaded NPs. **A** Z-Average hydrodynamic diameter, **B** ζ-potential, **C** loading efficiency and encapsulation efficiency of LIRA for each LIRA-loaded NPs; **D** TEM and FESEM images; **E** In vitro LIRA releases in pH 2.0 HCl solution for 2 h to simulate the gastric environment and in pH 7.4 PBS solution for 46 h to simulate the intestinal environment

Due to the high percentage of hydrophobic amino acid residues, zein is insoluble in pure water but soluble in 60–95% ethanol. The LIRA-loaded NPs were prepared by anti-solvent method. LIRA, CA, and RLs were synchronously embedded in the zein aggregates through hydrophobic interaction. The NPs without CA or RLs were also prepared as comparisons. The particle size of LIRA/CA@Zein/RLs was 160 nm while the absence of RLs would result in an obvious increase in particle size to 240 nm for LIRA/CA@Zein (Fig. 2 A), the absence of CA would also augment the particle size but much milder, which was 196 nm for LIRA @Zein/RLs. After 21-day storage, the particle size of LIRA/CA@Zein had grown by 159% but the size of LIRA/CA@Zein/RLs only had a 10% increase, indicating a stabilizing effect of the amphiphilic RLs (Additional file 1: Fig. S2). The encapsulation efficiencies of CA and RLs in LIRA/CA@Zein/RLs were 54.5% and 72.6%, respectively (Additional file 1: Fig. S3). Due to the carboxyl group’s negative charge in the molecule at pH 7.4, RLs drastically reduced the ζ-potential of NPs, suggesting that they had bound to the surface of nanoparticles (Fig. 2B). The ζ-potential alteration of LIRA@Zein/RLs also implied the existence of CA on the nanoparticle surface and possibly brought benefits for transportation by the bile acid route. The encapsulation efficiencies of LIRA in the three NPs were all above 75%, indicating an efficient entrapment (Fig. 2 C). The formation of LIRA/CA Complex slightly decreased the encapsulation efficiency of LIRA which was likely caused by the dissociation of LIRA oligomers. Electron microscopy images captured by transmission electron microscope (TEM) and field emission scanning electron microscopy (FESEM) of the three NPs in Fig. 2D show a spherical shape and well dispersion.

### In vitro release behaviors

The release behaviors of loaded LIRA from the three NPs were similar in simulated gastrointestinal fluids (Fig. 2E). In pH 2.0 HCl solution, about only 18% LIRA was diffused during the first 2 h. The cumulative releases of LIRA from LIRA/CA@Zein/RLs, LIRA@Zein/RLs, and LIRA/CA@Zein reached 58.4–67.4%, and 71.2% in the first 12 h, respectively. These results may be related to the existence of CA or RLs on the NP surface which not only brought the stabilizing effects but also hindered the peptide diffusion. A sustained release of LIRA lasting 48 h from LIRA/CA@Zein/RLs was observed in pH 7.4 PBS solution.

### In vitro cellular uptake

In vitro absorption evaluations were based on human epithelial colorectal adenocarcinoma Caco-2 cells, which are commonly used for oral absorption studies. To assess the potential effects caused by the cytotoxicity of NPs, the three zein-based NPs with various concentrations were incubated with Caco-2 cells for 48 h. The all above 90% cell viability reflected that our NPs were non-toxic to Caco-2 cells (Additional file 1: Fig. S4).

**Fig. 3:**
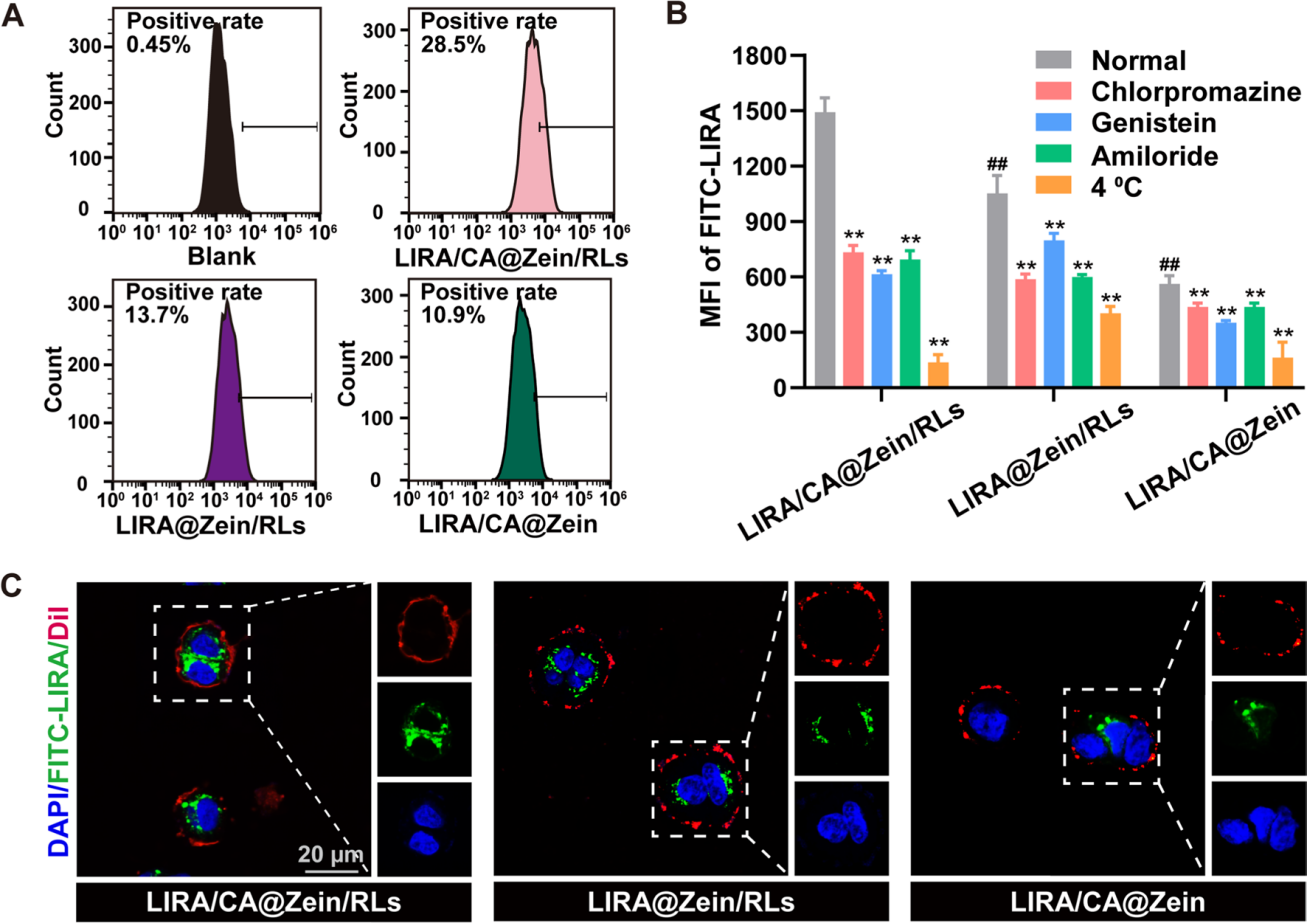
Cholic acid and rhamnolipids accelerated in vitro cell uptake of NPs. **A** Representative flow cytometry analysis of the uptake of FITC-LIRA/CA@Zein/RLs, FITC-LIRA@Zein/RLs, and FITC-LIRA/CA@Zein by Caco-2 cells after 2 h incubation. **B** Investigation of endocytic routes of the three NPs by mean fluorescence intensity (MFI) of FITC-LIRA after incubation with endocytosis inhibitors or in low temperature. **C** Representative CLSM images of FITC-LIRA loaded NPs incubated with Caco-2 cells. Blue: nuclei stained with DAPI, green: FITC-LIRA, red: cell membrane stained with Dil. ##P < 0.05, compared with LIRA/CA@Zein/RLs group; **P < 0.01, compared with the normal group for the same nanoparticle (n = 3)

Intracellular FITC-LIRA intensity from NPs was evaluated by flow cytometry and presented in Fig. 3 and Additional file 1: Fig. S5. FITC-LIRA/CA@Zein/RLs was superior in cell uptake rate as shown in Fig. 3 A. The mean fluorescence intensity (MFI) of FITC-LIRA in LIRA/CA@Zein/RLs group was 1.4-fold and 2.6-fold as compared to LIRA@Zein/RLs and LIRA/CA@Zein, respectively (Fig. 3B). The endocytosis of LIRA/CA Complex alone was limited (Additional file 1: Fig. S5). To clarify the influence factors on the cell uptake of NPs, we investigated the involved endocytic routes of the three NPs. By incubation under low temperature (4 °C) or with clathrin-mediated endocytosis inhibitor (chlorpromazine, CPZ), caveolae-mediated endocytosis inhibitor (genistein), and macropinocytosis inhibitor (amiloride) [31, 32], as shown in Fig. 3B, endocytosis of LIRA/CA@Zein/RLs was significantly inhibited under all the four conditions. At 4 °C, a 92% decrease of MFI was observed and attributed to the inhibition of energy-dependent process. Meanwhile, the endocytosis mediated by clathrin, caveolae, and micropinocytosis declined by 51%, 59%, and 53%, proving that multiple endocytosis pathways were involved for LIRA/CA@Zein/RLs. As for LIRA@Zein/RLs, its endocytosis was less dependent on the caveolae-mediated route and energy consumption, which may be ascribed to the absence of CA and resultant unavailability to the absorption route through the bile acid transporter. Besides, the close contact of CA with LIRA was critical, compared with LIRA/CA@Zein/RLs, the addition of CA in the RLs aqueous solution during the preparation of LIRA@Zein/RLs didn’t obtain the same endocytosis acceleration effect (Additional file 1: Fig. S5), which implied the importance of formation of LIRA/CA Complex in advance. According to the previous study, cell uptake is also a size-dependent process, particles with a diameter > 200 nm were barely involved in clathrin-coated pits [33]. Without the stabilizing effects of RLs, LIRA/CA@Zein exhibited larger particle size and was hindered during cell internalization. The confocal laser scanning microscope (CLSM) images in Fig. 3 C depicted the distribution of FITC-LIRA (green) within the Caco-2 membrane (red), thus confirming the endocytosis of FITC-LIRA-load NPs instead of adsorption on the cell membrane.

### Trans-mucus and transcellular permeability study

**Fig. 4:**
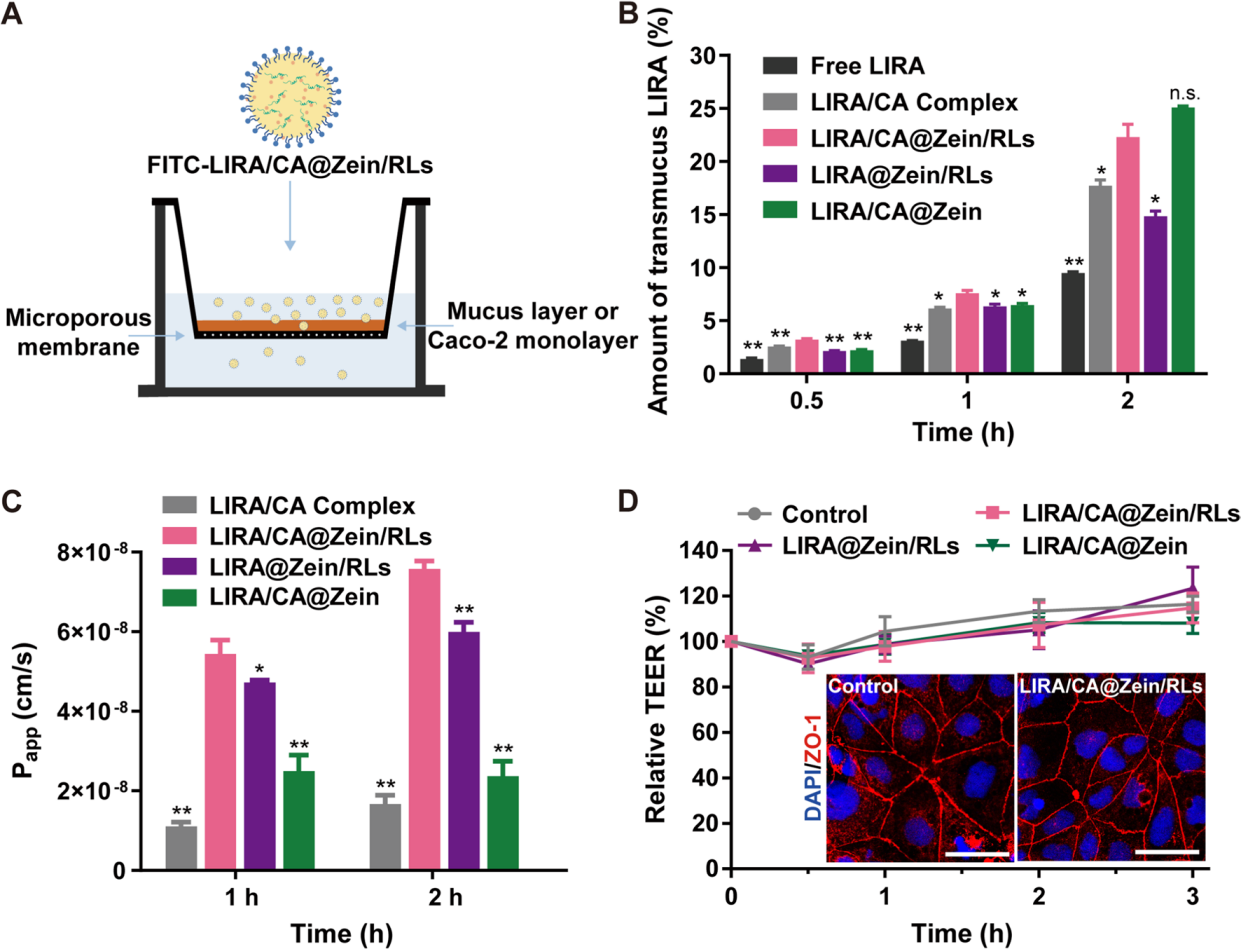
Trans-mucus and Caco-2 monolayer permeability of NPs. **A** Scheme of the experiments conducted in transwell^®^. **B** Tranmucus rate of LIRA from different formulations across simulated mucus layer composed of mucin. **C** P_app_ values of LIRA for LIRA/CA complex and different NPs in Caco-2 cell monolayers. **D** Relative TEER changes and ZO-1 immunofluorescence imaging of Caco-2 cell monolayers, the NPs were removed after 2 h incubation. Scale bar was 50 μm. *P < 0.05, **P < 0.01, n.s., no significance, compared with the LIRA/CA@Zein/RLs group (n = 3)

Mucus on the intestinal epithelial surfaces is capable of trapping and removing foreign particles. Rapid penetration through mucus opens significant opportunities for drug absorption by epithelium [34, 35]. Here, the mucus layer composed of 1% mucin solution was constructed to evaluate the mucus penetration ability of free LIRA, LIRA/CA Complex, and three NPs. To ensure integrity, the thickness of the mucus layer was about 600 μm in our experiment, which was thicker than that of 10−200 μm in the human body (Fig. 4 A) [36]. As shown by the transportation rate in Fig. 4B, LIRA/CA Complex and the three NPs had better mucus permeability than the free LIRA, indicating the encapsulation of LIRA in the complex or NPs could reduce the hydrophobic interaction of mucin with the palmitic acid group of LIRA. At 2 h, the superior transmucus ability of LIRA/CA@Zein/RLs than LIRA@Zein/RLs was observed which was likely related to the mucus glycoprotein depletion ability of CA [19].

After passing through the mucus, permeability through the epithelium was the main obstacle to absorption. The Caco-2 cell monolayer is a commonly acknowledged model to evaluate the intestinal absorption of drugs in vitro [37, 38]. The apparent permeability values (P_app_) of the three NPs and LIRA/CA Complex were consistent with the cell uptake results (Fig. 3 A), where both CA and RLs have positive effects on improving LIRA permeability (Fig. 4C). During the entire process of the experiment, none of the TEER (transepithelial electrical resistance) values of these groups changed significantly compared to the control group. Besides, as one of the vital tight junction-related proteins, the ZO-1 immunofluorescence in Fig. 4D confirmed the consecutive ZO-1 distribution between adjacent Caco-2 cells after LIRA/CA@Zein/RLs treatment. These results of unchanged TEER values and ZO-1 staining indicated an unimpaired tight junction, therefore, both CA and RLs transported LIRA across the Caco-2 cell monolayer by promoting transcellular rather than a paracellular pathway. As epithelial permeability enhancers, RLs were reported to effectively increase the paracellular and transcellular transport of macromolecules [27]. But in our experiment, only the transcellular route was observed which could be explained by the low concentration of RLs used (80 µg/mL) and that about 70% of RLs (Additional file 1: Fig. S2) were in NPs rather than free in the solution.

### LIRA/CA@Zein/RLs enhanced intestinal permeability

**Fig. 5:**
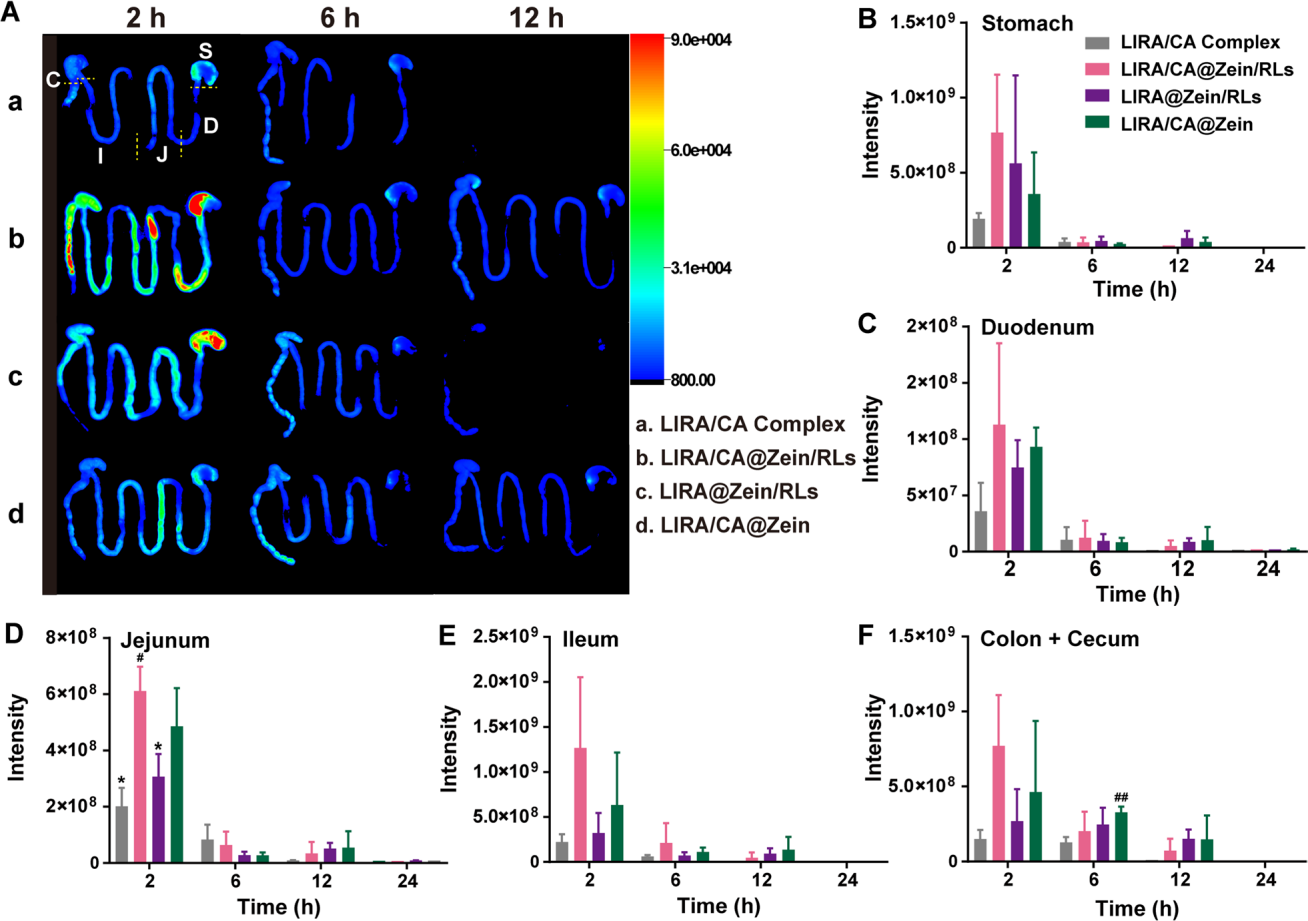
The distribution of Cy5-LIRA in GIT of mice. **A** Representative images of murine GIT after oral administration with LIRA/CA Complex, LIRA/CA@Zein/RLs, LIRA@Zein/RLs, and LIRA/CA@Zein, respectively (the intensity at 24 h was weak and not shown here); Analysis of the Cy5-LIRA fluorescence intensity in **B** stomach, **C** duodenum, **D** jejunum, **E** ileum, and **F** cecum and colon, *P < 0.05 compared with LIRA/CA@Zein/RLs group, #P < 0.05 and ##P < 0.01 compared with LIRA/CA Complex group (n = 3)

To observe the bio-distribution of LIRA in vivo, Cy5-LIRA/CA Complex or Cy5-LIRA-Loaded NPs were orally administrated. The distribution of Cy5-LIRA in the gastrointestinal tract and organs was visualized ex vivo at various time intervals. The fluorescence signals of the gastrointestinal tract (GIT) were strongest at 2 h for all groups, as shown in Fig. 5 A–F, and gradually waned over time. Without protection, the LIRA/CA Complex’s signal quickly faded (Fig. 5 A). Quantitative analysis of GIT segments revealed that, after 2 h digestion, more LIRA/CA@Zein/RLs were retained in the small intestine, but only in the jejunum did it demonstrate significant differences from LIRA/CA Complex and LIRA@Zein/RLs (Fig. 5D). It should be related to the permeability enhancement caused by CA and RLs because the unabsorbed NPs would be digested and eliminated quickly.

**Fig. 6:**
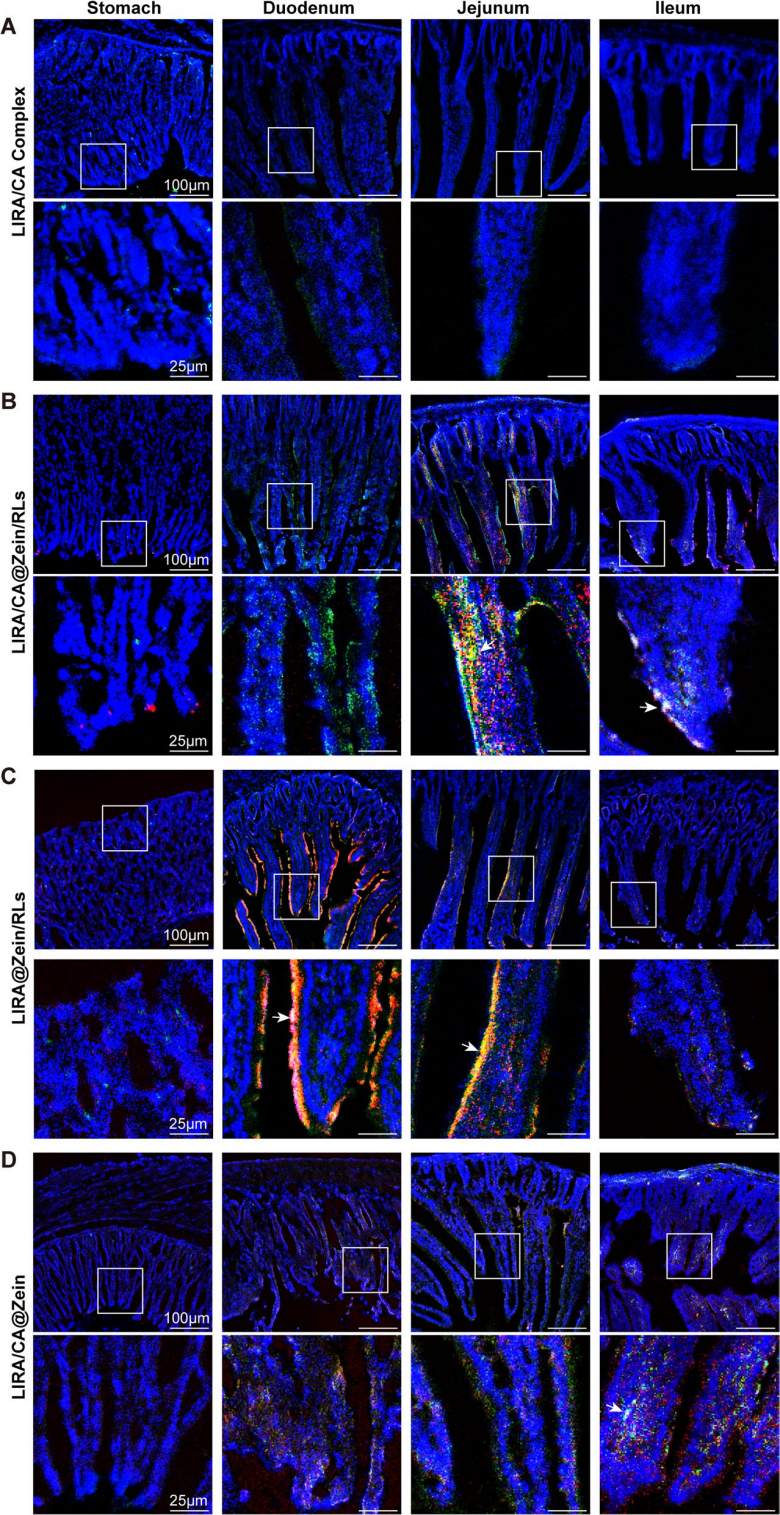
Representative CLSM images of FITC-LIRA and RITC-zein in stomach, duodenum, jejunum, and ileum sections after oral administration with **A** LIRA/CA Complex, **B** LIRA/CA@Zein/RLs, **C** LIRA@Zein/RLs and **D** LIRA/CA@Zein for 2 h, respectively. Arrows represent FITC-LIRA colocalized with RITC-zein. Blue: nuclei, green: FITC-LIRA, red: RITC-zein

To further observe the permeability enhancement in various segments of the GIT, sections were prepared and visualized by CLSM. The time point was set after oral administration for 2 h when the FITC-LIRA signal was strongly presenting along the GIT. Due to fast degradation, LIRA/CA Complex had only a few fluorescence signals in stomach and scarce signals along the GIT (Fig. 6A). LIRA/CA@Zein/RLs showed bright fluorescence deep into the intestinal villi and mucosa in duodenum, jejunum, and ileum. The fluorescence signals of FITC-LIRA and RITC-zein were partly overlapped, revealing both intact and dissociated NPs existed during the absorption process (Fig. 6B). LIRA@Zein/RLs had comparable fluorescence intensity in the intestine to LIRA/CA@Zein/RLs but exhibited markedly different fluorescence locations, most LIRA@Zein/RLs NPs only accumulated in the mucus, presenting poor transepithelial efficiency without the assistance of CA (Fig. 6C). This result was consistent with the widely reported fact that CA could accelerate the absorption of NPs by ASBT (apical sodium-dependent bile acid transporter) and IBABP (ileal bile acid-binding protein) expressed in the intestine [18, 39]. As for LIRA/CA@Zein, the fluorescent intensity in the microvilli was weak, suggesting an indistinctive transepithelial flux after losing the stabilizing and possible membrane perturbation effects of RLs (Fig. 6D). Consequently, CA and RLs cooperatively guaranteed the effective transcytosis and intestinal villi absorption of LIRA/CA@Zein/RLs in the intestine.

### LIRA/CA@Zein/RLs improved the absorption into the systemic circuit

**Fig. 7:**
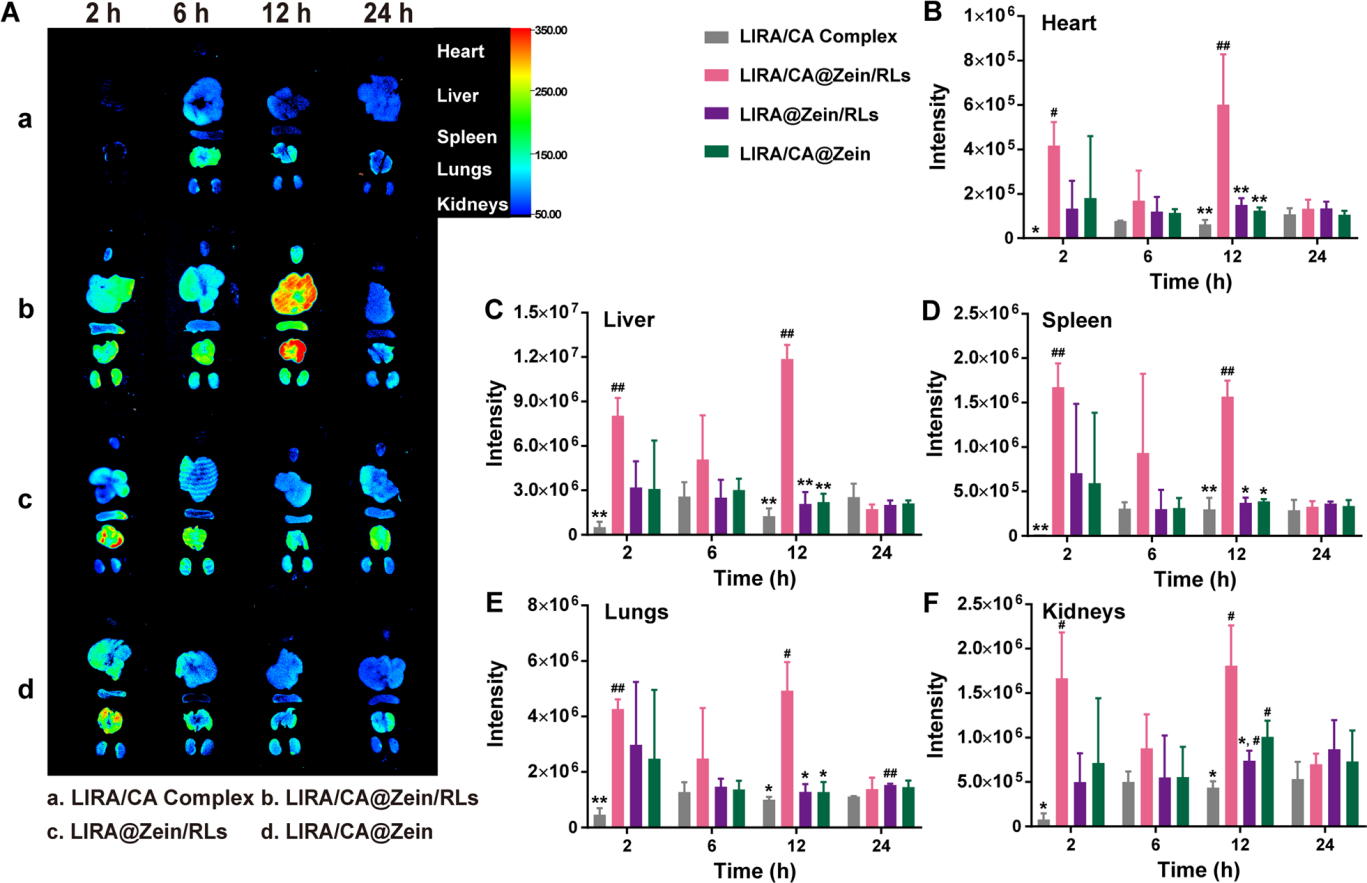
The distribution of Cy5-LIRA in main organs. **A** Representative images of murine main organs after oral administration with LIRA/CA Complex, LIRA/CA@Zein/RLs, LIRA@Zein/RLs and LIRA/CA@Zein; Analysis of the Cy5-LIRA fluorescence intensity in **B** heart, **C** liver, **D** spleen, **E** lung, and **F** kidney, *P < 0.05 and **P < 0.01 compared with LIRA/CA@Zein/RLs group, #P < 0.05 and ##P < 0.01 compared with LIRA/CA Complex group (n = 3)

The organ biodistribution of the LIRA/CA Complex and NPs was presented in Fig. 7A. LIRA/CA Complex exhibited a weak fluorescence signal within 24 h, indicating limited oral absorption. All the fluorescence of the three NPs formulations remained for over 24 h in the organs including the liver, spleen, lungs, and kidney. The quantitative analysis in Fig. 7B–F showed that LIRA/CA@Zein/RLs exhibited the strongest fluorescence intensity in this process, and at the time of 12 h, the LIRA signal significantly increased by 3.0- and 3.8-fold in heart, 4.7- and 4.4-fold in liver, 3.2- and 3.0-fold in spleen, 2.8- and 2.9-fold in lungs, 1.4- and 0.8-fold in kidney as compared to LIRA/CA@Zein and LIRA@Zein/RLs, indicating the cooperative absorption acceleration brought by CA and RLs was still obvious after oral delivery in vivo.

### Hypoglycemic efficacy of LIRA/CA@Zein/RLs in type 2 diabetic mice

**Fig. 8:**
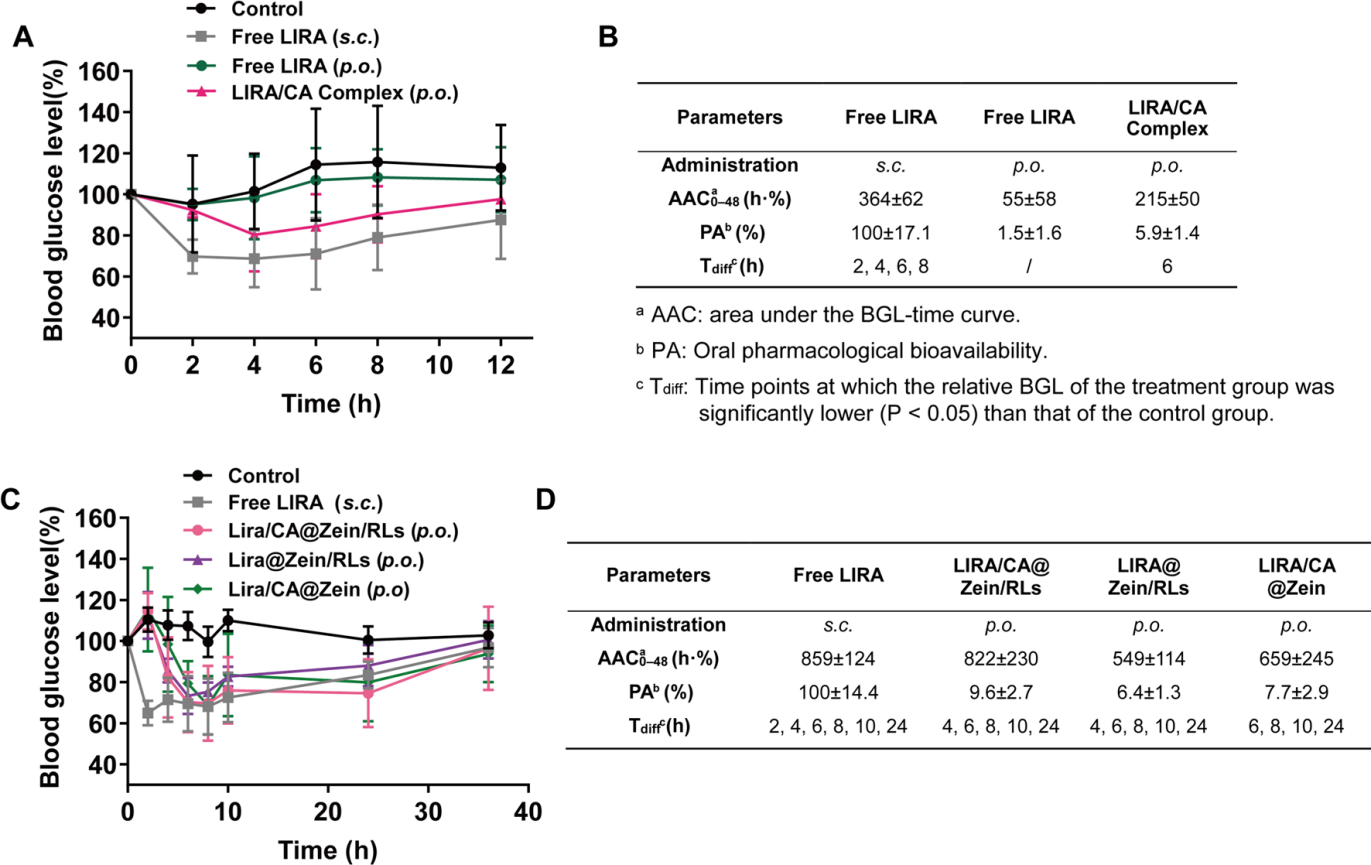
Oral hypoglycemic efficacy in Type 2 Diabetic Mice. **A** Relative BGL changes and **B** pharmacodynamic data after oral administration with LIRA/CA Complex, Free LIRA solution, and saline at a LIRA oral dose of 2 mg kg^− 1^ and subcutaneous injection dose of 0.2 mg kg^− 1^; **C** Relative BGL changes and **D** pharmacodynamic data after oral administration with LIRA/CA@Zein/RLs, LIRA/CA@Zein and LIRA@Zein/RLs at a LIRA oral dose of 4 mg kg^− 1^ and subcutaneous injection dose of 0.4 mg kg^− 1^ (n = 6)

To evaluate the hypoglycemic effects of different formulations, pharmacodynamics experiments on T2DM mice were conducted. The dosage of orally administered LIRA formulations was 10 times higher than *s.c.* administered LIRA, considering the enzymatic degradation and absorption rate in GIT. [40] Results in Fig. 8 A, B revealed that the oral free LIRA didn’t show any hypoglycemic effect but LIRA/CA Complex (oral) group had an obvious hypoglycemic effect as reflected by the T_diff_ points. But due to the fast degradation, the oral pharmacological bioavailability (PA) of LIRA/CA Complex was limited to 5.9%.

The efficacy of the three NPs was evaluated. As shown in Fig. 8C, D, LIRA/CA@Zein/RLs and *s.c.* LIRA presented comparable abilities in BGL control, and a remarkable hypoglycemic effect was observed over 24 h. The oral pharmacological bioavailability of LIRA/CA@Zein/RLs was 9.6%, whereas the PA values of LIRA/CA@Zein and LIRA@Zein/RLs which did not contain CA or RLs were 6.4% and 7.7%, respectively. The 33.3% and 19.8% reduction in bioavailability caused by the removal of CA or RLs indicated that both CA and RLs are essential for promoting the in vivo absorption of LIRA.

## Conclusion

Efficient delivery of LIRA by LIRA/CA@Zein/RLs into the intestinal epithelium was found in the GIT and comparable oral hypoglycemic effects with the subcutaneous injection group were observed in T2DM mice. The specific non-covalent interaction between LIRA and CA resulted in the formation of LIRA/CA Complex which showed enhanced trans-mucus and trans-epithelial efficiency. Besides, zein/RLs NPs prepared by an easy anti-solvent method not only achieved a high LIRA loading efficiency but also obtained good physical stability and this stabilizing effect brought by RLs didn’t show the unfavorable effect on endocytosis that commonly encountered by hydrophilic coatings. Attributed to the absorption acceleration of both CA and RLs, LIRA/CA@Zein/RLs efficiently penetrated into the intestinal villi, thereby achieving lasting hypoglycemic effects in vivo. Our results demonstrated an extraordinarily promising oral delivery system of LIRA.

## Materials and methods

### Materials

Zein, cholic acid, and rhamnolipids R90 (90%) were purchased from Sigma-Aldrich (Shanghai, China). Liraglutide was from GL Biochem (Shanghai). Mucin (from porcine stomach) was from meilun bio (Shanghai, China). Sulfo-cyanine 5 NHS ester (Cy5, analytical grade) was from Lumiprobe (Maryland, USA). Fluorescein isothiocyanate (FITC) was from Tokyo Chemical Industry (Tokyo, Japan). Anthrone was from Aladdin (Shanghai, China). Chlorpromazine, genistein, amiloride, and 1, 1′-dioctadecyl-3,3,3′,3′-tetramethylindocarbocyanine perchlorate (Dil) were from Beyotime Biotechnology (Shanghai, China). All cell culture reagents and materials were from Thermo Fisher Scientific (Shanghai, China). All other reagents were from Sinopharm Chemical Reagent (Shanghai, China). Total bile acid (TBA) kits were from Nanjing Jiancheng Bioengineering Institute.

### Preparation and characterization of LIRA/CA complex

LIRA was dissolved in 20 mM pH 7.4 phosphate buffer. CA, RLs, and SDS were dissolved in deionized water separately and adjusted to pH 7.4. LIRA solution was mixed with cholic acid solution at a molar ratio of 1:18 or 1:36 (LIRA: CA). Similarly, LIRA solution was mixed with RLs or SDS solution at a molar ratio of 1:18 (LIRA: RLs/SDS). The final concentration of LIRA was kept at 1 mg mL^− 1^. The mixed solution was stirred at room temperature for 10 min under magnetic stirring.

The free LIRA and LIRA mixed with CA, RLs or SDS were loaded to the upper chamber of ultrafiltration spin columns (Millipore Amicon**®** Ultra, cutoff molecular weight: 50 kDa) and centrifuged at 12,000 rpm for 20 min, the amount of LIRA that is ultrafiltered across the membrane was analyzed by BCA assay. After lyophilization, FTIR spectra of LIRA, CA, physical mixture of LIRA and CA, and LIRA/CA Complex were recorded on a FTIR spectrometer (Nicolet 6700, Thermo Nicolet) using potassium bromide tableting.

### Molecular docking by autodock vina

The structure of LIRA in aqueous solution was obtained from Protein Data Bank whose code was 4apd1. AutoDock Tools 1.5.7 were employed to add hydrogens to LIRA structure and to generate the input files of ligands with rotatable bonds. The grid box with dimentions of x: 80 Å, y: 45 Å and z: 50 Å, centering on x: − 0.675, y: − 0.006 and z: 0.348 was built to include the whole LIRA structure. Ligand-LIRA docking was calculated using Lamarckian Genetic Algorithm by Autodock Vina 1.1.2. [41, 42] Docking of various ligands produced the affinity values for comparison, the non-covalent interaction in the calculation comprises hydrogen bonds, hydrophobic force, Gaussian steric interactions, repulsion and torsion terms and the best docking conformation between ligand and LIRA with the highest binding affinity was extracted for analysis by PyMol software.

### Fabrication and characterization of LIRA/CA complex-loaded zein nanoparticles

LIRA/CA Complex was prepared as described above. Zein was dissolved in ethanol/water (9:1, V/V). The above LIRA/CA Complex solutions and zein solution were mixed to form an ethanol/water stock solution. Subsequently, the RLs aqueous solution was added into the mixed solution under magnetic stirring and stirred at room temperature for 3 h before supplementing deionized water to the final volume. The final concentrations of LIRA, CA, zein and RLs were 0.5, 1.0, 5.0 and 0.8 mg mL^− 1^. Due to the hydrophobicity of zein, the LIRA/CA Complex-loaded zein NPs was obtained. The formulations that did not contain CA or RLs were prepared as a control.

Fluorescein isothiocyanate (FITC)-conjugated liraglutide (FITC-LIRA) was synthesized and the loading efficiency (LE) of LIRA was determined by ultrafiltration method as previously reported [20]. The ultrafiltered FITC-LIRA was analyzed by a microplate reader (Cytation, Biotek), CA was analyzed by total bile acid kits and RLs were quantified by colorimetric determination of sugars with anthrone sulfate [43]. The laser particle size analyzer (Anton Paar) was used to analyze hydrodynamic diameter (Dh) and ζ-potential. The Morphology was observed by TEM (CM120, Philips) and FESEM (Ultra 55, Zeiss).

The in vitro releases of LIRA from NPs were evaluated in the dialysis tubing (cutoff molecular weight 100 kDa, Spectrum Laboratories Inc.) as reported in our previous study [20]. The simulated gastrointestinal fluid was pH 2.0 HCl solution at 2 h and 10 mM pH 7.4 phosphate buffer during 2–48 h.

### In vitro mucus penetration study

The mucus layer was simulated by the mucin solution of 10 mg mL^−1^. A transwell® plate (12-well, PET, 1.0 μm, Millipore) was used and the mucus layer thickness in the apical side (AP) was 0.6 mm. The FITC-LIRA or FITC-LIRA-loaded NP solution was gently added on the top of the mucus layer and incubated in the basolateral side (BL) with fresh HBSS at 37 °C. Solution in the BL was collected and analyzed at predetermined time intervals. The transportation rate is calculated by the ratio of FITC-LIRA in BL to the total.

### Cytotoxicity measurements

Caco-2 cells were seeded in 96 well plates at a density of 5000 cells/well. After overnight incubation, the original medium was replaced with serum-free fresh medium containing gradient concentrations of NPs. After incubation at 37 °C for 48 h. The medium was removed and 3-(4,5- dimethylthiazol-2-yl)-2,5-diphenyltetrazolium bromide (MTT) was added at a concentration of 0.5 mg mL^− 1^. The incubation continued for 4 h, then the MTT solution was replaced by dimethyl sulfoxide. After shaking for 10 min, the absorbance was measured at 490 nm.

### Cellular uptake

To evaluate the uptake behavior, FITC-LIRA was used in the preparation of NPs. Caco-2 cells were seeded in 24 well plates at a density of 5 ⋅ 10^4^ cells/well and allowed to adhere for 24 h. Then, free FITC-LIRA, LIRA/CA Complex, and FITC-LIRA loaded NPs (50 µg mL^−1^) were added. After 2 h incubation, cells were digested and resuspended in 250 µL Hank’s Balanced Salt Solution (HBSS). The fluorescence intensity in cells was measured by flow cytometer (FACS Calibur, BD).

The influence of CA and RLs on cell uptake was observed by CLSM. Briefly, Caco-2 cells were seeded in confocal dishes. After 24 h, NPs (50 µg mL^−1^) were added and incubated for 2 h. Then cells were fixed and cell membrane were stained with Dil after nucleus staining with DAPI.

To evaluate the endocytosis mechanism of NPs, Caco-2 cells in 24 well plates were prepared and measured with the same procedure as above, but pre-incubated with different endocytic inhibitors which were 10 µg/mL chlorpromazine (CPZ), 100 µM genistein and 10 µg/mL amiloride for 0.5 h before addition of NPs solutions.

### Permeability in caco-2 cell monolayers

Caco-2 cell monolayers were constructed as previously reported. Briefly, Caco-2 cells were seeded in 24-well transwell® (PET, 1.0 μm, Millipore) at a density of 2 ⋅ 10^4^ cells/well and cultured for 14−21 days. A transepithelial electrical resistance (TEER) over 500 Ω·cm^2^ was necessary. Before the addition of FITC-LIRA loaded NPs solutions (50 µg/mL), the medium in the apical side (AP) and basolateral side (BL) was replaced with pre-warmed HBSS and the cell monolayers were incubated for 30 min. At predetermined time intervals, the FITC-LIRA concentration in the BL solution was analyzed by a microplate reader. The apparent LIRA permeability (P_app_) was calculated by

$${\text{P}}_{\text{a}\text{p}\text{p}}=\frac{\text{Q}}{\text{A}{\text{C}}_{0}\text{t}} (\text{n}=3)$$

where *Q* was the accumulative amount (ng) of FITC-LIRA in the BL, *A* was insert membrane growth area which was 0.33 cm^2^ in the 24-well transwell®, *C*_*0*_ was the initial FITC-LIRA concentration (ng/mL) in the AP and *t* was the duration time (s).

TEER values at different time points were also measured in the experiment [44]. To further validate the intactness of tight junctions, the Caco-2 cells on the microporous membrane of transwell® were cut down and incubated with the primary antibody against ZO-1 (1:400, ab150083, Abcam), the corresponding fluorescent secondary antibodies (1:400, 21773-1-AP, proteintech) were incubated after three washes.

### The organ distribution of LIRA after oral administration

Healthy male C57BL/6 mice aged 6–8 weeks were purchased from the Animal Center of the Chinese Academy of Sciences (Beijing, China). The animal experiments were performed at Experimental Animal Center of School of Pharmacy of Wenzhou Medicine University, and were approved by Experimental Animal Ethics Committee of School of Pharmacy of Wenzhou Medicine University (ID Number: wydw2021-0140). We synthesized Sulfo-cyanine-5 NHS ester labeled LIRA (Cy5-LIRA) and Rhodamine B isothiocyanate-conjugated zein (RITC-zein) as reported [45], followed by the preparation of double fluorescence-labeled NPs. The mice were fasted overnight with free access to water and randomly divided into four groups. The solutions of LIRA/CA Complex, LIRA/CA@Zein/RLs, LIRA@Zein/RLs, and LIRA/CA@Zein were administrated orally at a dose of 0.4 mg kg^−1^. After 2, 6, 12, and 24 h, the mice were sacrificed and organs were taken out, washed, and imaged (In Vivo Xtreme, Bruker).

### Oral pharmacodynamics evaluation in T2DM mice

The streptozotocin-induced T2DM mice were built as reported. [46] The T2DM mice with fasting blood glucose levels (BGL) higher than 16 mM were chosen to evaluate the hypoglycemic effect of formulations. The experiment was carried out in two batches. In the first batch, the mice were randomly divided into 4 groups and fasted for 10 h with free access to water, Free LIRA and LIRA/CA complex were orally administrated at a dose of 2 mg kg^−1^, respectively; LIRA solution was subcutaneously injected at a dose of 0.2 mg kg^−1^, saline was orally administered as a control. In the second batch, the mice were randomly divided into 5 groups and fasted as above. The mice in LIRA/CA@Zein/RLs, LIRA@Zein/RLs, and LIRA/CA@Zein groups (Control, *p.o.*) were administrated orally at a dose of 4 mg kg^−1^, and saline was administrated (Saline, *p.o*.) as control. In the Free LIRA group, LIRA solution (LIRA, *s.c.*) was subcutaneously injected at a dose of 0.4 mg kg^−1^. At predetermined time intervals, BGL was measured based on blood from the tail by a glucometer (ACCU-CHEK Active, Roche). Food was provided for 2 h at 12, 24, and 36 h post-administration, and water was available throughout the experiment. The oral pharmacological bioavailability (PA) of LIRA was calculated according to the following equation:

$$\text{P}\text{A} \left(\text{\%}\right)=\frac{{(\text{A}\text{A}\text{C}}_{\text{N}\text{P}\text{s}, p.o.}-{\text{A}\text{A}\text{C}}_{\text{C}\text{o}\text{n}\text{t}\text{r}\text{o}\text{l}})/{\text{D}\text{o}\text{s}\text{e}}_{\text{N}\text{P}\text{s}, p.o.}}{{(\text{A}\text{A}\text{C}}_{\text{L}\text{I}\text{R}\text{A}, s.c.}-{\text{A}\text{A}\text{C}}_{\text{C}\text{o}\text{n}\text{t}\text{r}\text{o}\text{l}})/{\text{D}\text{o}\text{s}\text{e}}_{\text{L}\text{I}\text{R}\text{A},s.c.}}\times 100\text{\%} (\text{n}=6)$$

where *AAC* was the area above the relative BGL-time curve.

### Statistical analysis

All the data were presented as mean ± standard deviation (SD). Statistical significance was analyzed by one-way ANOVA analysis followed by Turkey post hoc tests, and P < 0.05 was regarded to be statistically significant.

## Supplementary Information

**Additional file 1: Figure S1.** The images and affinity values of other docking conformations between CA and LIRA. **Figure S2.** The Z-Average hydrodynamic diameter change of NPs after 21-day storage at 2 ~ 8 °C. **Figure S3.** Encapsulation of cholic acid and rhamnolipids in LIRA/CA@Zein/RLs. **Figure S4.** Caco-2 cell viabilities after 48 h incubations with the three NPs. **Figure S5.** The impact of CA on the Caco-2 cell uptake.

## Data Availability

All data generated or analyzed during this study are included in this published article and its supplementary information file.
