# Supplementary material for: Intestinal epithelium penetration of liraglutide via cholic acid pre-complexation and zein/rhamnolipids nanocomposite delivery
Source: J Nanobiotechnology. 2023 Jan 16;21:16. doi: 10.1186/s12951-022-01743-9 (PMC9843898; doi:10.1186/s12951-022-01743-9)
Supplement: Supplementary file 1 — Additional file 1: Figure S1. The images and affinity values of other docking conformations between CA and LIRA. Figure S2. The Z-Average hydrodynamic diameter change of NPs after 21-day storage at 2 ~ 8 °C. Figure S3. Encapsulation of cholic acid and rhamnolipids in LIRA/CA@Zein/RLs. Figure S4. Caco-2 cell viabilities after 48 h incubations with the three NPs. Figure S5. The impact of CA on the Caco-2 cell uptake. [file 12951_2022_1743_MOESM1_ESM.docx]

**Additional file 1**

**Intestinal epithelium penetration of liraglutide via cholic acid pre-complexation and zein/rhamnolipids nanocomposite delivery**

Xiaoyan Bao,^1^‡ Kang Qian,^2^‡ Mengjiao Xu,^1^‡ Yi Chen,^3^ Hao Wang,^1^ Ting Pan,^1^ Zhengyi Wang,^1^ Ping Yao^4^* and Li Lin^1^*

^a^ School of Pharmaceutical Sciences, Wenzhou Medical University, Wenzhou 325035, China

^b^ Key Laboratory of Smart Drug Delivery, Ministry of Education, School of Pharmacy, Fudan University, Shanghai 201203, China

^c^ State Key Laboratory of Functions and Applications of Medicinal Plants, Guizhou Medical University, Guiyang 550014, China

^d^ State Key Laboratory of Molecular Engineering of Polymers, Collaborative Innovation Center of Polymers and Polymer Composite Materials, Department of Macromolecular Science, Fudan University, Shanghai 200438, China.

‡ These authors contribute equally

* Correspondence authors

E-mail address: [linliwz@163.com](mailto:linliwz@163.com) (Li Lin); Tel: +86 135 8759 8702

E-mail address: [yaoping@fudan.edu.cn](mailto:yaoping@fudan.edu.cn) (Ping Yao); Tel: +86 137 6163 8566

**Supporting Figures**


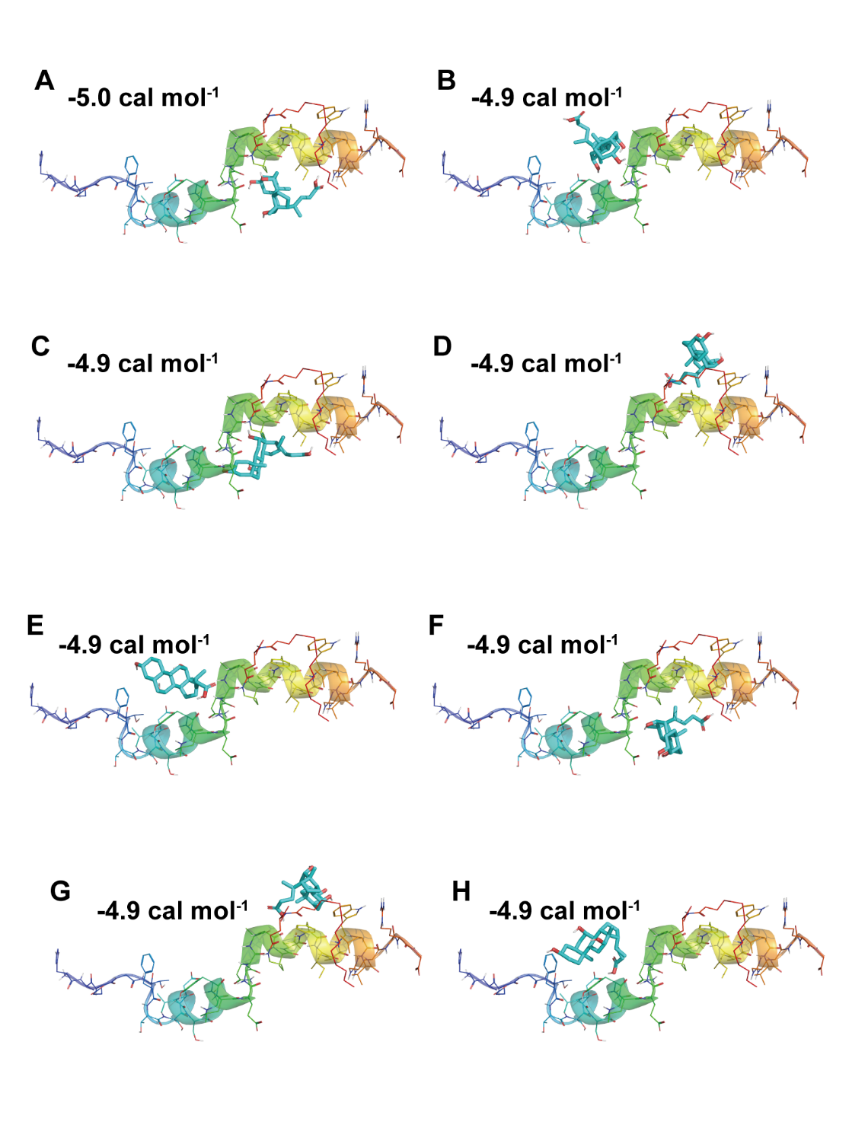


**Fig. S1** (A-H) The images and affinity values from second to ninth rank of the docking conformations between cholic acid and liraglutide.


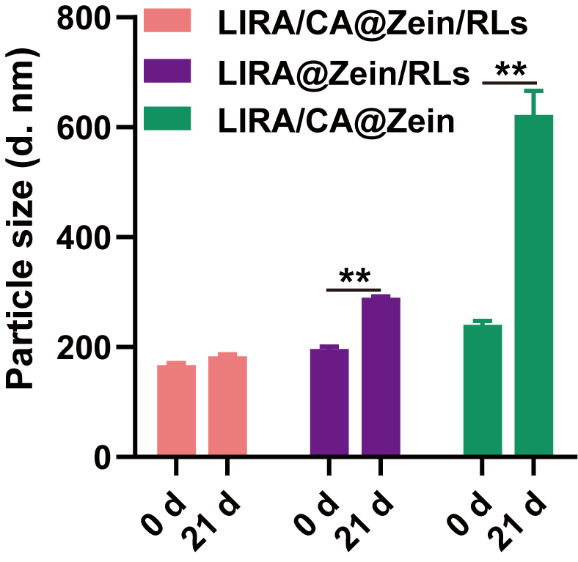


**Fig. S2** The Z-Average hydrodynamic diameter change of NPs after 21-day storage at 2 ~ 8 °C, **P < 0.01 compared with the particle size in 0 d (n = 3).

**Fig. S3** Encapsulation of cholic acid and rhamnolipids in LIRA/CA@Zein/RLs (n = 3).

**Fig. S4** Caco-2 cell viabilities after 48 h incubations with LIRA/CA@Zein/RLs, LIRA @Zein/RLs and LIRA/CA@Zein at the zein concentrations of 16−500 μg/mL (n = 6).


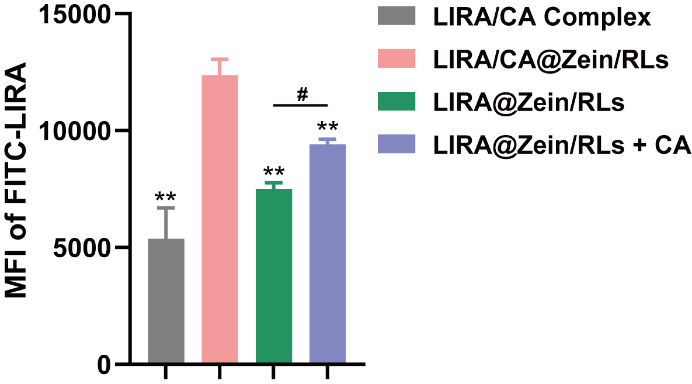


**Fig. S5** The impact of CA on the Caco-2 cell uptake. Mean fluorescence intensity of FITC-LIRA in LIRA/CA@Zein/RLs, LIRA@Zein/RLs and mixture of LIRA@Zein/RLs and CA solution groups determined by flow cytometry. ** P < 0.01, compared with the LIRA/CA@Zein/RLs group; # P < 0.05, compared with LIRA @Zein/RLs group (n = 3).
